# Are Non-Coding RNAs Useful Biomarkers in Parathyroid Tumorigenesis?

**DOI:** 10.3390/ijms221910465

**Published:** 2021-09-28

**Authors:** Cinzia Aurilia, Simone Donati, Gaia Palmini, Francesca Miglietta, Irene Falsetti, Teresa Iantomasi, Maria Luisa Brandi

**Affiliations:** 1 Department of Experimental and Clinical Biomedical Sciences, University of Florence, Viale Pieraccini 6, 50139 Florence, Italy; cinzia.aurilia@unifi.it (C.A.); simone.donati@unifi.it (S.D.); gaia.palmini@unifi.it (G.P.); francesca.miglietta@unifi.it (F.M.); irene.falsetti@unifi.it (I.F.); teresa.iantomasi@unifi.it (T.I.); 2 Fondazione Italiana Ricerca sulle Malattie dell’Osso (FIRMO Onlus), 50141 Florence, Italy

**Keywords:** parathyroid glands, biomarkers, non-coding RNAs, miRNAs, long non-coding RNAs, circular RNAs

## Abstract

Tumors of the parathyroid glands are common endocrine diseases almost always characterized by parathyroid hormone hypersecretion that determines the clinical manifestations of primary hyperparathyroidism, such as fatigue, kidney problems, weakness, brittle bones, and other symptoms. Most parathyroid neoplasia are benign adenomas, although rare malignant forms have been described. They are heterogeneous in terms of clinical presentation and the associated signs and symptoms overlap with those of disease and aging. Furthermore, most patients with hypercalcemia are discovered during routine blood tests for other reasons. Surgical removal is considered the main therapeutic option to cure these endocrine tumors and, therefore, innovative therapeutic approaches are actively required. Recently, a growing number of studies have suggested that alterations to the epigenetic mechanisms could play a pivotal role in parathyroid tumorigenesis. Most of the attention has been focused on non-coding RNAs (ncRNAs) (i.e., miRNAs, lncRNAs, and circRNAs) whose expression profile has been found to be deregulated in parathyroid tumors. The aim of the present paper is to give an insight into the ncRNAs involved in parathyroid tumorigenesis, which could be used in the future either as innovative diagnostic biomarkers or as therapeutic targets for the treatment of this endocrine neoplasia.

## 1. Introduction

The parathyroid glands are four small endocrine glands located symmetrically in the neck, on the back of the thyroid lobes [1]. Although a majority of the population has four parathyroid glands, there are cases of supernumerary glands, and some cases of three glands have been reported [2]. Classically, it is possible to discriminate two superior and two inferior parathyroids [3]. The first are located between the demarcation of the recurrent laryngeal nerve and the inferior thyroid artery and the cricothyroid ligament; meanwhile, inferior parathyroids are situated between the junction of the inferior thyroid vein and the inferior thyroid artery, at the posterior aspect of the thyroid. However, it is possible to find one or more parathyroid glands residing in other locations, such as the retro-esophageal area, the lateral neck, and the mediastinum, which are referred to as ectopic parathyroids. Histologically, we find two cell types which compose these glands, the chief cells and oxyphil cells. While chief cells appear smaller and are responsible for the secretion of parathyroid hormone (PTH), oxyphil cells are morphologically larger, their number seems to reduce with age, and their role has not yet been fully understood [4].

The function of the parathyroids is to regulate serum calcium levels. In fact, when the levels of this ion in the blood are low, the parathyroid glands secrete PTH, which, acting on various organs (i.e., kidneys, intestines, and bones), restores the correct levels of the ion itself. On the contrary, an increase in calcium levels in the blood inhibits the production and release of PTH from the parathyroids [5].

Parathyroid dysfunctions are classified in two categories: hyperparathyroidism and hypoparathyroidism. The first is caused by an increased function of the parathyroids and PTH secretion, while hypoparathyroidism is due to a decreased activity of parathyroid cells and a consequent lower release of the hormone. Hyperparathyroidism condition is often due to the existence of a parathyroid adenoma (PA), or alternatively to a diffuse four-gland hyperplasia, or malignancy. However, malnutrition, vitamin D deficiency, chronic kidney disease, and other types of tumors may also lead to hyperparathyroidism occurrence [6,7]. Hypoparathyroidism, on the other hand, could be due to a simple failure of the parathyroid glands function, or to an accidental removal of the same glands during an extensive neck surgery or, finally, to the treatment with radiotherapy for tumors in the surrounding area, which causes the destruction of the parathyroid glands [4,8]. The etiology of these tumors remains unknown for many patients, but it has been observed that sporadic forms of adenoma are commonly related to mutations in the cyclin D1/PRAD1 gene, while the hereditary forms have been found in families affected by diseases, such as Multiple Endocrine Neoplasia type 1 or 2 (MEN 1 or 2) and Familial Hypocalciuric Hypercalcemia (FHH) [4].

Regarding diagnosis, most patients with PAs are asymptomatic, and their discovery occurs accidentally through routine laboratory tests, as, for example, the total calcium blood test, through which is possible to reveal a condition of hypercalcemia. If the hypercalcemia persist further exams are necessary, such as 25-hydroxyvitamin D, PTH, creatinine and 24-h urinary calcium measurement, which can be useful in excluding secondary causes of hyperparathyroidism [9,10,11,12].

Parathyroid imaging (i.e., ultrasound, scintigraphy, single-photon emission computerized tomography (SPECT), magnetic resonance imaging (MRI), and four-dimensional CT) has no utility for supporting or excluding a diagnosis of PA even though it is frequently used to provide an accurate localization of adenomatous glands thus identifying those patients who should be candidates for minimally invasive parathyroidectomy [12]. Nevertheless, diagnostic imaging is much less accurate in evaluating multiglandular parathyroid gland disease [13].

In some cases, very high serum levels of calcium and PTH may be signs of parathyroid cancer, which manifests itself with these clinical features in only about 1% of patients. However, there is not a clear threshold of values to discriminate malignancy and, therefore, it continues to be difficult to make a differential diagnosis between adenoma and parathyroid cancer [14,15]. Immunohistochemistry may be useful in the classification and diagnosis of parathyroid tumors, and in particular parafibromin immunohistochemical expression. Inactivating mutations of the *cell division cycle protein 73 homolog* (*CDC73*) gene that encodes parafibromin, an evolutionarily conserved tumor suppressor protein whose loss of expression has been implicated in parathyroid tumorigenesis, have been reported up to 70% of sporadic PCs, whereas they are rarely detected in benign adenomas [16,17]. However, the limited number of studies and the caveats due to the high variability in parafibromin loss makes its assessment not straightforward, complicating the diagnosis of PC.

Generally, for patients with moderate serum calcium levels and symptoms, or patients for whom surgery is not indicated or who have failed previous surgical intervention, a more conservative approach is preferred, which includes biochemical assessment and targeted medical intervention; on the contrary, patients with high serum PTH and calcium levels, presenting all the severe complications that this entails, require surgical approach with parathyroidectomy [18,19].

In recent years, it has been observed that epigenetics is involved in several endocrine neoplasia, including parathyroid tumorigenesis [20]. 

Epigenetics is a set of intracellular mechanisms that can modulate gene expression without making changes to the DNA sequence, which includes acetylation and methylation of histone proteins, methylation of cytosine, and the action of non-coding RNAs (ncRNAs) at post-transcriptional level [21]. 

In particular, ncRNAs are classified according to the number of nucleotides that make them up. Short ncRNAs consist of fewer than 200 nucleotides, including Piwi-interacting RNAs (piRNAs), small nuclear RNAs (snRNAs), small nucleolar RNAs (snoRNAs), small interfering RNAs (siRNAs), and microRNAs (miRNAs). NcRNAs that are longer than 200 nucleotides are referred to as long non-coding RNAs (lncRNAs) [22]. In addition, the latter can be divided into two major categories of intragenic and intergenic lncRNAs, based on their genomic position [21,23,24,25,26]. Finally, it has also been observed that some types of lncRNAs are able to create a covalent bond between their own 3′ and 5′ ends, thus forming the class of circular lncRNAs (circRNAs) [24]. 

Different ncRNAs have several mechanisms of action. In particular, and of interest for this review, miRNAs regulate gene expression by interacting principally with 3′-UTR of mRNA target, by taking it to degradation or by preventing its translation [27]. LncRNAs can act in various ways, but generally they perform their function by acting as competitive endogenous RNAs (ceRNAs), by binding their miRNA targets and inhibiting the action of the latter [21]. 

Regarding circRNAs, these molecules can either act as ceRNAs or they can be translated into functional micropeptides [28]. 

The aim of this review is to provide an overview of miRNAs, lncRNAs, and circRNAs involved in parathyroid tumorigenesis, which could be considered as future diagnostic biomarkers and therapeutic targets for this disease.

In order to fulfil this purpose, we carried out a systematic literature search on PubMed database by employing different combinations of relevant terms, including “parathyroid glands”, “parathyroid adenomas”, “non-coding RNAs”, “miRNAs”, “lncRNAs”, and “circRNAs”. All relevant studies released between 2010 and 2021 were selected and reviewed.

## 2. Differentially Expressed miRNAs in Parathyroid Tumors

In this section we will review seven studies that investigate on the miRNA expression profile in parathyroid carcinomas (PCs) to efficiently distinguish between malignant and benign tumors of parathyroid glands.

In the first study, Rahbari et al. [29] compared miRNA expression profiles in 9 PC, 12 PA, and 15 parathyroid hyperplasia to four pooled normal parathyroid gland samples, derived from bioptic sample tissues obtained during a minimally invasive parathyroidectomy, as described by the authors. miRNA expression profile has been analyzed by using microarray system analysis, identifying, respectively, 167, 277, and 157 differentially expressed miRNAs as compared to controls. Based on adjusted *p*-value < 0.01, the expression levels of 24 miRNAs were deregulated among PC and PA groups, but only 13 were commercially available and assayed by quantitative real-time PCR (qPCR) (i.e., miR-26b, miR-27a, miR-27b, miR 30b, miR-28, miR-34a, miR-100, miR-126, miR-126*, miR-145, miR-423-3P, let-7a, and let-7f). Similar to what they observed in microarray, the levels of three out 13 selected miRNAs were significantly more reduced in carcinoma than in adenoma samples (miR-26b, miR-30b, and miR-126*). Among these, miR-126* showed the highest degree of accuracy as a predictive biomarker for distinguishing PA from carcinoma in receiver operating characteristics (ROC) analysis (area under the curve (AUC) = 0.776). Furthermore, target prediction analyses revealed a potential target for miR-28, *CDC73* gene, but no correlation was observed between their expression levels in PC samples.

In the wake of the previous study, Sadowski et al. [30] tried to get possible novel insights on the molecular mechanisms of parathyroid tumorigenesis, by analyzing the transcription pattern of 29 candidate genes associated with parathyroid gland function, cell cycle, and apoptosis, and circadian clock in formalin-fixed paraffin-embedded samples derived from an individual with adenoma, sporadic primary, and secondary hyperparathyroidism using NanoString technology and RT-PCR. For their analysis, the authors selected significantly upregulated miRNAs in sporadic primary hyperparathyroidism adenoma from previous analyses of Rahbari et al. [29]. Moreover, these selected miRNAs were simultaneously predicted to target different genes, including rearranged during transfection (RET), with c-MET, vitamin D receptor (VDR), and calcium-sensing receptor (CaSR), which were found downregulated in snap-frozen samples during Nanostring analysis. However, no significant differences in miRNA expression (i.e., miR-21-5p, miR-29b-3p miR-144-3p, miR-211-5p, miR-218-5p, and miR-330-3p) were observed following qPCR validation. This result could be due either to the small number of samples or to the interindividual variability in each group. Collectively, these data suggest possible novel molecular mechanisms underlying different pathologies related to parathyroid glands, though further analyses with a larger number of samples will be needed to draw conclusions regarding the possible role of miRNAs in parathyroid tumorigenesis. 

The aim of a study performed by Corbetta et al. [31] was to identify the miRNAs differentially expressed in PCs harboring *CDC73* inactivating mutations and in normal parathyroid tissue. Among the 279 miRNAs analyzed, 3 and 14 miRNAs resulted in significant over and down expression in PCs; specifically, miR-222 and miR-503 were upregulated and miR-139 and miR-296 were downregulated, suggesting their potential roles as oncogenes or oncosuppressors, respectively. They also observed that the expression levels of miR-222, miR-296, and miR-503 could be used to discriminate PAs from PCs, while miR-139 was downregulated in the same way in both tumor types. Finally, it was also seen that the expression levels of miR-222 and miR-296 in PAs were negatively correlated, respectively, with the expression levels of p27/kip1 and the hepatocyte growth factor receptor-regulated tyrosine kinase substrate (HGS) mRNAs. In conclusion, these data indicate that there is an altered expression of miRNAs in PCs, and especially of miR-296, which might play a crucial role as an oncosuppressor in parathyroid tumors.

Additionally, a study by Vaira et al. [32] analyzed the expression of a subset of miRNAs belonging respectively to the two clusters on chromosome 19, C19MC (i.e., MIR519C, MIR521, MIR520G, MIR512-3p, MIR515-3p, MIR522, MIR519D, MIR517A, MI518E, MIR520H, MIR520D-3p and MIR523) and MIR-371-3 (i.e., MIR371 and MIR372), which have been associated with stem cell biology and tumorigenesis. They observed no significant difference in their expression between PAs and normal parathyroid gland samples, while the expression profile of C19MC and miR_371-3 cluster members was able to differentiate between PAs and PCs. MIR517C was among those that showed the most significant difference and whose levels positively correlated with parathyroid hormone (PTH), serum calcium, and tumor weight. Furthermore, they studied the mechanism underlying C19MC cluster activation, finding that copy number variations in the chr.19q13.41–42 region were associated with MIR517C overexpression. Regarding the methylation status of C19MC promoter, they observed a reduction in C19MC promoter methylation compared to normal parathyroid glands, where it was methylated. In conclusion, their findings revealed that alterations of C19MC cluster are distinctive to carcinoma with respect to adenoma parathyroid tumors and the potential oncogenic role of chromosome 19 miRNA clusters on parathyroid tumorigenesis. 

Given the relevance of these miRNA clusters in parathyroid tumorigenesis, the same research group [33] explored the role and underlying mechanism of miR-372 in primary parathyroid tumor cultures. They confirmed previous data, establishing that the miR-372 levels were higher in half of PAs and in most atypical PAs and PCs, both with respect to normal parathyroid glands. In PAs, miR-372-positive parathyroid cells were scattered throughout all tumor parenchyma. Because p21 and the large tumor suppressor kinase 2 (LATS2) are known cell cycle-related target genes of miR-372, they investigated their relationship. Their findings revealed that miR-372 ectopic overexpression inhibited their expression, both at mRNA and protein levels. However, even though the viability was unaffected through this miRNA modulation, a reduction in camptothecin-induced apoptosis was observed in parathyroid cells. Finally, they obtained insight into the regulation of miR-372 with parathyroid-associated genes, such as TBX1, GCM2, PTH, and WNT. TBX1 and GCM2 gene expression was not affected by miR-372 mimic transfection, while its overexpression positively correlated with PTH levels and reduced the activation of the Wnt signaling pathway via the upregulation of Wnt-inhibitor DKK1. Overall, these results indicated that miR-372 could play an important role in the parathyroid, thus providing novel possible insights into the stratification and treatment of patients.

Overall, the altered expression of the miRNAs belonging to C19MC and MIR371-3 clusters might distinguish between the PC and PA, but further analyses in larger cohorts are needed to validate such analysis.

Hu et al. [34] analyzed miRNA expression patterns between tissue samples derived from 17 PCs and 41 sporadic PAs. Levels of miR-222 were significantly upregulated, while those of miR-30b, miR-126*, miR-139, and miR-517c were significantly reduced in PCs compared with PAs. Results from ROC analysis showed that the combination of miR-30b and miR-139 was the most powerful predictive biomarker for differentiating between PCs and PAs. Spearman’s rank correlation revealed that intact PTH (iPTH), serum calcium, and alkaline phosphatase levels were negatively correlated with the expression profile of miR-30b. These findings suggest that the combination of miR-30b and miR-139 could be a promising diagnostic strategy for PCs. Further studies are needed in larger and independent patient cohorts before achieving definitive evidence.

Recently, on the basis of the emerging concept of exosomal miRNAs, Wang et al. [35] investigated, for the first time, the differential expression of miRNAs in serum exosomes derived from PCs and adenoma patients, the latter being used as controls. Five exosomal miRNAs (i.e., miR-27a-5p, -miR-93-5p, miR-134-5p, miR-146b-5p, and miR-381-3p) were found to be upregulated in the serum of four PC patients with respect to four PA patients by using next-generation sequencing (NGS) screening. These results have been validated using qPCR, where only exosomal miR-27a-5p levels were significantly increased in the serum of individuals affected by PCs, as well as the ability to discriminate between PC patients and controls with an AUC of 0.8594. The expression of the five above-mentioned miRNAs has been further verified in carcinoma and adenoma parathyroid-derived tissues. The results obtained display a similar trend to those in exosome, showing an increase in miR-27a-5p, miR-93-5p, and miR-381-3p in the carcinoma tumor types.

Collectively, these seven studies analyzed here provided novel insight into differentially expressed miRNAs by tumor types, suggesting them as potential biomarkers which could be used as helpful molecules for distinguishing PAs from PCs.

Analyzing the literature on the research about miRNA expression profiles for distinguish PAs from PCs, it’s important to also evaluate studies performed on MEN1 syndrome, since MEN1 patients develop endocrine tumors, especially of parathyroid glands, pancreas, and pituitary. MEN1-related parathyroid lesions are usually benign, and only rarely have cases of parathyroid cancer been described. 

Luzi et al. [36] were the first to demonstrate that MEN1 parathyroid tumorigenesis could be ascribed to epigenetic mechanisms, where miR-24-1 acting as an oncomiRNA through the binding to the 3′UTR of menin could control the onset and progression of this disease via a negative regulatory loop. In particular, they evaluated the expression levels of MEN1 mRNA and menin protein, and miR-24-1, in eight MEN1 parathyroid adenoma tissues (four without and four with loss of heterozigosity (LOH) of MEN1 gene), three non-MEN1 PA tissues, and one normal parathyroid gland tissue. Their analysis revealed an absence of expression of miR-24-1, MEN1 mRNA, and menin in MEN1 PAs tissues with LOH, while MEN1 parathyroid samples from patients preserving the wild type allele of the MEN1 gene exhibited a reduced expression of MEN1 mRNA, miR-24-1 overexpression, and markedly low levels of menin compared with sporadic forms and normal parathyroid gland. Overall, their findings suggest that the expression of this miRNA only in MEN1 parathyroid adenoma tissues without LOH could be responsible for parathyroid tumorigenesis previous to the MEN1 LOH occurrence.

Later, the same research group [37] studied the global miRNA expression profile in parathyroid tissues derived from MEN1 patients to try to understand their possible role in MEN1 parathyroid tumorigenesis. They found that, of the 1890 miRNAs analyzed in LOH and non-LOH MEN1 samples, two miRNAs were deregulated in LOH MEN1 PAs and eight in non-LOH MEN1 PAs, compared with control samples. In addition, six other miRNAs were also observed to be differentially expressed between MEN1-LOH and non-LOH MEN1 PAs. After validation, by using RT-PCR, the most differentially expressed miRNAs were miR-1301, miR-664, and miR-4258. Furthermore, in silico analyses indicated some genes involved in parathyroid tumorigenesis as possible targets of miR-664 and miR-1301. Thus, three miRNAs involved in the development of parathyroid tumors in MEN1 patients were identified, which could be used as possible diagnostic and prognostic biomarkers of this pathology.

Recently, to overcome the difficulties in discriminating sporadic and hereditary parathyroid tumors, Hwang et al. [38] determined whether a miRNA signature could differentiate these parathyroid tumor types. A microarray-based analysis was initially performed to identify differentially expressed miRNAs between sporadic and MEN1-associated parathyroid tumors compared to healthy parathyroid tissues. According to the adjusted *p*-value controlling for a false discovery rate (FDR) < 0.05, 10 differentially expressed miRNAs were identified between parathyroid tumor samples compared with controls. Following validation by qPCR analysis in independent samples consisting of 25 sporadic and 12 hereditary parathyroid tumors, and 24 normal parathyroid tissues, only miR-199b-5p was significantly found downregulated and negatively correlated with PTH levels in the sporadic tumors and upregulated in the hereditary forms compared with its expression in normal tissues. Furthermore, ROC curve analysis confirmed its diagnostic relevance in discriminating between sporadic and hereditary tumor types with an AUC value of 0.863, with a specificity of 100% and a sensitivity of 67%, suggesting its role as potential biomarker in parathyroid tumors.

Based on these findings, these four miRNAs (i.e., miR-24, miR-1301, miR-664, and miR-4258) which have been potentially associated with MEN1 parathyroid tumorigenesis could be possible targets for the future development of innovative therapeutic approaches. In addition, miR-199-5p could be considered as a promising biomarker to discriminate the hereditary form from sporadic parathyroid tumor.

In closing, Yavropoulou et al. [39] evaluated the expression of specific miRNAs in serum and tissue samples obtained from patients with sporadic parathyroid adenoma (sPAs) compared to the control population. At the tissue level, among the miRNAs selected on the basis of their interaction with genes involved in parathyroid tumorigenesis, the expression levels of miR-17-5p, miR-135b-5p, miR-31-5p, miR-186-5p, and miR-330-3p were significantly downregulated, while those of miR-24-3p and miR-29b-3p were significantly upregulated in sPAs versus the control group. In addition, they found that miR-135b-5p expression was also decreased in the serum of sPA patients compared to controls. Finally, it was also discovered that the miRNAs involved in the regulation of genes related to parathyroid tumorigenesis, such as CaSR (miR-135b-5p, miR-31-5p), MEN1 (miR-24-3p, miR-29b-3p), cyclin-dependent kinase inhibitors (miR-186-5p), cyclin D1 (miR-17-5p), and β-catenin (miR-330-3p), had lower expression levels in sPAs compared to healthy parathyroid gland tissue. Thus, these data suggest an important involvement of epigenetics in the development and progression of parathyroid tumors.

In Table 1, we have summarized the differentially expressed miRNAs, which we have analyzed in this section.

## 3. Differentially Expressed lncRNAs in Parathyroid Tumors

In the last years, lncRNAs, regulatory ncRNAs, have become the subject of particular interest in the scientific world. Thanks to their ability to regulate gene expressions, they are being studied to understand their role in the pathogenesis of several diseases [40], including tumors [41]. Recent studies showing their involvement in tumorigenesis are highlighting how important these molecules would be not only in the development of future antineoplastic therapies, but also how they could be used as tumor biomarkers [42]. Therefore, in this section we will make an update on the state of the art of the research on the role of lncRNAs in parathyroid tumors biology.

In their study, Jiang et al. [43] conducted an expression analysis of lncRNAs to distinguish PA from PC samples. The lncRNA profile analyzed by microarray revealed 1809 differentially expressed lncRNAs between parathyroid neoplasms. Subsequently, the use of RT-PCR identified four statistically differentially expressed lncRNAs (lnc-RP11-1035H13.3.1-2:1, lnc-FLT3-2:2, lnc-FEZF2-9:2, and LINC00959) between the two types of parathyroid tumors. Thus, the deregulation of these lncRNAs might lead the scientific community to consider these molecules as potential biomarkers to discriminate PAs from carcinomas and possibly to use them as future therapeutic targets.

In addition, Yu et al. [44] described the discovery of useful biomarkers to characterize the different tumor lesions of the parathyroid glands. Besides confirming the diagnostic value of chromogranin A and PTH in parathyroid neoplasms, they performed an expression profile analysis of several lncRNAs, including HOX transcript antisense intergenic RNA (HOTAIR), long intergenic non-protein coding regulator of reprogramming (Linc-ROR or ROR), and metastasis-associated lung adenocarcinoma transcript one (MALAT1), analyzed by using RT-PCR and in situ hybridization assays. The data obtained showed that ROR was found to be downregulated during disease progression, suggesting that it may act as an oncosuppressor in parathyroid neoplasms. Therefore, this finding could lead ROR to being considered as a possible therapeutic target and prognostic marker for parathyroid tumorigenesis. 

Similarly, Morotti et al. [45] investigated the expression profile of 90 known lncRNAs in normal, adenomatous, and carcinomatous parathyroid tissues and confirmed deregulation of 11 of them. The expression of these lncRNAs was correlated with the status of CDC73 and MEN1 genes, cytogenetic aberrations, or clinical features. In fact, they have shown that LOH at chromosomes 1, 11, 15, 21, and 22 led to a deregulation of lncRNA expression in PAs. In particular, they observed that PAs with MEN1 gene mutations showed an increased expression of six lncRNAs (BC200, HAR1B, HOXA3as, NEAT1, SNHG6, and ZFAS1) compared to PAs with wild-type MEN1 genes. In the same way, PCs with mutations in the CDC73 gene overexpressed BC200 lncRNA compared to CDC73 wild-type carcinomas. These findings suggest that the oncosuppressors MEN1 and CDC73 could be considered as potential modulators of the expression of different lncRNAs. These latter findings could help in the further understanding of the phenotypic diversity of PCs and PAs, as well as providing new possible personalized therapies.

In 2019, Zhang et al. [46] studied the expression of mRNAs and lncRNAs in six PAs, six PCs, and four normal parathyroid tissues. The results show that 2165 mRNAs and 2641 lncRNAs were differentially expressed between PAs and PCs. Subsequent Gene Ontology (GO) and KEGG analyses demonstrated that deregulated mRNAs were mainly involved in the energy metabolism and extracellular matrix (ECM)-receptor interaction pathways. In addition, the authors selected seven lncRNAs and three mRNAs, previously found to be deregulated, for validation by in situ hybridization and RT-PCR methods, discovering that lncRNA PVT1 and GLIS2-AS1 were significantly deregulated in PCs and PAs. In conclusion, this study identified two lncRNAs as possible new markers for discriminating PAs and PCs.

Since the research in parathyroid tumor on lncRNAs is in its beginning stages, more experiments are needed to validate the differential expression of these lncRNAs, which have been identified in the above-mentioned studies in order to adopt them as biomarkers for typifying the different parathyroid lesions, in association with those already in use in the clinical context. 

In Table 2 we have summarized differentially expressed lncRNAs that have been validated to date as targets in parathyroid tumors.

## 4. Differentially Expressed circRNAs in Parathyroid Tumors

Next to miRNAs and lncRNAs, we find another class of ncRNAs called circRNAs. These latter molecules have diverse molecular functions, and for this reason they have a role in neural system, cardiovascular system, and also in cancer [47]. Hence, in relation to their capacity to regulate gene expression, circRNAs are involved in the development of several diseases, especially in cancer [48]. Precisely, in light of the emerging role of circRNAs in carcinogenesis, here we decided to analyze the state of the art of the role of circRNAs in parathyroid tumors.

It was 2018 when Yavropoulou and his group [49] described the evaluation of the circRNA profile in six sporadic PAs and four normal parathyroid samples. Through the use of microarray hybridization assay, the authors found that 35 circRNAs were reported to be differentially expressed between these two groups. Specifically, it was observed that 13 circRNAs were downregulated and 22 were upregulated. Among them, four upregulated (cirRNA_402533, cirRNA_051778, cirRNA_0008267 and cirRNA_406174) and two downregulated circRNAs (circRNA_058097 and circRNA_032603) showed a higher fold-change value, >5-fold and >3-fold, respectively. Subsequent validation by qPCR revealed that five circRNAs were significantly altered with fold-change values comparable to those obtained by microarray analysis. Moreover, further investigation showed that there was a difference in the expression of circRNAs in male and female patients. In fact, they observed that nineteen circRNAs were upregulated and four were downregulated in male vs. female patients. 

Similar work was carried out by Hu et al. [50] who analyzed the expression profile of circRNAs in six PAs, six PCs, and four normal parathyroid tissues. They found that 5310 and 1055 circRNAs were differentially expressed in PCs and PAs compared to healthy tissues, respectively. Subsequently, they constructed a circRNA–mRNA co-expression network in order to try to identify the critical circRNAs in PC. In total, six circRNAs (circ_0079278, circ_0035563, circ_0017545, circ_0085534, circ_0001687 and circ_0075005) and four host genes (FSCN1, AKR1C3, ANXA2, and MYC) were selected for qPCR validation. The results showed that only four circRNAs (circRNA_0017545, circRNA_0035563, circRNA_0075005, and circRNA_0001687) and four mRNAs (MYC, AKR1C3, FSCN1, and ANXA2) were differentially expressed between PA and PC tissues. Additional studies revealed a significant positive correlation between circ_0035563 and ANXA2 mRNA, circ_0079278 and FSCN1 mRNA and between circ_0017545 and AKR1C3 mRNA, but not between circ_0085534 and MYC mRNA. Finally, the ROC analysis highlighted that circ_0075005 and MYC were the best promising markers to distinguish PC from benign lesions with an AUC of 0.770 and 0.909, respectively.

As can be seen, research studies on the presence and the role of circRNAs in parathyroid tumors started only a little more than three years ago. Therefore, further studies are need to know these molecules and their functions to think to use them as future potential diagnostic and prognostic biomarkers in clinical practice of parathyroid tumors.

In Table 3 we have summarized the differentially expressed circRNAs that have been validated to date as targets in parathyroid tumors.

## 5. Discussion

Parathyroid glands are important organs of the endocrine system that control the levels of calcium and phosphorus, and bone mineralization, through the release of PTH [51]. 

Uncontrolled parathyroid cell proliferation may lead to the development of benign and, rarely, malignant tumor forms [52].

These tumors are most often associated with PTH overproduction, determining the clinical features of primary hyperparathyroidism (pHPT). pHPT is the third most common endocrine disorder, following thyreopathies and diabetes [52], resulting from a single parathyroid adenoma in 85% of patients. In the remaining cases, the underlying pathological process is either a consequence of multiglandular parathyroid disease (multiple/hyperplasia adenomas) or, rarely, derives from PCs.

Typically, surgery is considered the gold standard for the treatment of patients affected by parathyroid tumors, because pharmacological therapy provides relief only from the symptoms of hypercalcemia, without impacting the disease’s progression [20]. 

Therefore, a better understanding of the molecular mechanisms underlying parathyroid tumorigenesis could lead to the identification of novel biomarkers that may improve the diagnosis and treatment of these tumors, for which the only current treatment option for patients is surgery. As some studies showed a deregulation of miRNA expression pattern in PCs, such as the members of C19MC and MIR371-373 clusters, and that the parafibromin loss could potentially be the result of epigenetic mechanisms involving miRNAs, further insight into parathyroid tumorigenesis could pave the way towards the possible use of these molecules as a helpful adjunct for the pre-existing markers, such as parafibromin and Ki-67, for discriminating between PCs and PAs. In fact, a very difficult debate on the differential diagnosis of these parathyroid tumors is still taking place.

During recent years, a large number of epigenetic studies have shown that these mechanisms are often altered in human tumors [53]. Epigenetics is the study of hereditable and reversible phenotypic variations that do not involve a change in the DNA sequence. This includes DNA methylation, histone modifications, and regulation of gene expression by ncRNAs [54]. 

It is well known that different ncRNAs, including lncRNAs, circRNAs, and miRNAs, could crosstalk with each other in order to directly or indirectly regulate gene expression [55].

Based on this review, increasing evidence has shown that an aberrant expression of these molecules has been observed in parathyroid neoplasms with respect to normal parathyroid tissue, showing their relevant role in the tumorigenesis of this endocrine disorder. It has been observed that the deregulation of ncRNAs’ expression is involved in the tumorigenesis of several human organs. According to different findings, the expression profile comparison of miR-24, miR-126*, and miR-139 between human tumor and non-tumor tissues has been widely studied, revealing that a deregulation of these miRNAs could have a role in the tumorigenesis of several cancers, including breast, prostate, bladder, lung, and gastrointestinal tract cancers [56,57,58]. Here, the aforementioned miRNAs have been identified as potentially involved in parathyroid tumorigenesis in two independent studies. Unfortunately, the full understanding of miRNA regulation and function is limited by the fact that in vitro parathyroid cell models are extremely limited and remain hardly usable for transfection studies. Therefore, the issues of experiments designed to analyze the expression of candidate target genes being mainly performed on parathyroid tissues and the small sample size contribute to the fact that studies on miRNAs and their underlying mechanisms are still in their infancy. Even an aberrant expression of the lncRNAs NEAT1 and ROR has been documented in human tumor occurrence, such as breast, lung, ovarian, hepatocellular, and colorectal cancers [59,60]. Additional studies are needed to corroborate these data in order to produce consistent outcomes for their potential application as either therapeutic targets or as diagnostic and prognostic biomarkers in human tumors. Currently, different RNA-based strategies have been developed and could be used for the treatment of different diseases, including short hairpin RNAs (shRNAs), small interfering RNAs (siRNAs), antisense oligonucleotides (ASOs), ASO anti-microRNAs (antimiRs), miRNA sponges, therapeutic circRNAs, and miRNA mimics [61,62,63,64]. Increasing interest has been placed on the use of either miRNA inhibitors, to cause specific oncomiRs downregulation, or miRNA mimics, which are designed to increase the expression of a tumor suppressor miRNA, resulting in posttranscriptional or translational inhibition of the gene expression [65].

lncRNA- and circRNA-based therapeutics are also gaining interest for their clinical potential. In fact, both these molecules are being explored as potential biomarkers for a range of different diseases, even though no lncRNA-based therapies have entered clinical trials so far [65]. In this regard, the use of circRNAs as miRNA sponges represents a promising opportunity, but further investigation is needed to improve the comprehension of their regulatory mechanisms for their future use as biomarkers of parathyroid tumors [21,66].

The potential application of ncRNA-based therapies in the clinical field could bring benefits both to patients’ quality of life and to outcomes, thus reducing costs related to patients’ management. In particular, their use could guide each patient towards a tailored therapeutic treatment, to monitor drug response in PA patients receiving medications, such as calcimimetics and bisphosphonates, and also to assess the efficacy of the treatment. Further discoveries should be made and, in perspective, the usage of ncRNA-based methods as a possible therapeutic tool will require additional efforts to overcome some biological drawbacks related to their short half-life in an in vivo environment, the possible activation of innate immune response, and to avoid off-target effects.

Importantly, ncRNAs have also been described in different body fluids, and their concentrations have been closely associated with the physiological and pathological states of a patient [67]. 

Based on their readily accessible and minimally invasive properties, ncRNAs, and in particular miRNAs and lncRNAs, have emerged as promising diagnostic and prognostic biomarkers in a wide range of diseases [68,69,70]. Furthermore, these molecules show optimal biological properties, such as high stability against degradation, a long biological half-life, and easy detection through available techniques in laboratories, such as qPCR [68,71,72]. The combination of specific circulating miRNA (c-miRNA) expression patterns along with other biomarkers could improve the predictive and prognostic power of parathyroid neoplasms. Although lncRNAs are less stable than miRNAs, these long transcripts are also detectable in biological fluids [71]. However, further studies are required to determine the potential of circulating ncRNAs as reliable biomarkers for parathyroid-related diseases.

Furthermore, some critical issues have to be addressed before considering practical applications. One of the major challenges is the lack of an established control for normalizing c-miRNA data and, hence, quantifying their levels in biological fluids [73]. In addition, extensive preclinical studies should be carried out to establish c-miRNAs as biomarkers for these endocrine disorders.

Despite the increasing interest regarding the potential role of ncRNAs as possible biomarkers, research for their application as future diagnostic and prognostic tools for parathyroid tumors is still in its infancy, and further novel studies are necessary.

In conclusion, we give insight into the different ncRNA types associated with parathyroid tumorigenesis, which could be considered for either the future development of molecular ncRNA-based therapeutic strategies or the early diagnosis and prognosis of these tumors. Extensive studies investigating the cellular and molecular mechanisms of ncRNA function in the development and progression of parathyroid tumors are needed to translate their use from the laboratory bench to clinical practice as future diagnostic and prognostic tools for this neoplasm.

## Figures and Tables

**Table 1:** Summary of differentially expressed miRNAs in parathyroid tumors.

Candidate miRNAs	Expression Levels	miRNA Expression Profiling Platform	AUC Value	Study
miR-26b, miR-30b, and miR-126*	↓	miRNA arrays, qPCR	0.766 (miR-126*) for discriminating parathyroid adenoma from carcinoma	[29]
/	/	qPCR	/	[30]
miR-222, miR-503, miR-139, and miR-296	↑ and ↓	Microarray, qPCR	/	[31]
C19MC miRNAs and miR-372	↑	qPCR	/	[32]
miR-372	↑	qPCR	/	[33]
miR-222, miR-30b, miR-126*, miR-139, and miR-517c	↑ and ↓	qPCR	0.864 (miR-30b), 0.747 (miR-139), 0.888 (miR-30b + miR-139) for discriminating between PC patients and adenoma patients	[34]
Serum exosomal miR-27a-5p	↑	NGS, qPCR	0.8594 for discriminating PCs from adenoma	[35]
miR-24-1	↑	qPCR	/	[36]
miR-1301, miR-664, and miR-4258	↑ and ↓	Microarray, qPCR	0.65 (miR-4258), 0.84 (miR-1301), 0.92 (miR-664), 0.84 (miR-4258) for discriminating MEN1-LOH from MEN1-no-LOH PAs and control pool	[37]
miR-199b-5p	↓	Microarray, qPCR	0.863 for distinguishing between sporadic and hereditary parathyroid tumors	[38]
miR-17-5p, miR-135b-5p, miR-31-5p, miR-186-5p, miR-330-3p, miR-24-3p, and miR-29b-3p	↑ and ↓	Microarray, qPCR	/	[39]

↑, increase in miRNA expression levels; ↓, reduction in miRNA expression levels.

**Table 2:** Summary of differentially expressed lncRNAs in parathyroid tumors.

Candidate lncRNAs	Expression Levels	lncRNA Expression Profiling Platform	AUC Value	Study
LINC00959, lnc-FLT3-2:2, lnc-FEZF2-9:2, and lnc-RP11-1035H13.3.1-2:1	↓ and ↑	Microarray, qPCR	0.88 (global lncRNA score) for discriminating PCs from adenoma	[43]
ROR		In situ hybridization assay, qPCR	/	[44]
BC200, HAR1B, HOXA3as, NEAT1, SNHG6, and ZFAS1	↑	In situ hybridization assay, qPCR	0.74 (BC200) for discriminating PCs from adenoma and atypical adenomas, and for discriminating PCs with CDC73 mutation from wild-type carcinomas	[45]
PVT1 and GLIS2-AS1	↑ and ↓	Microarray, qPCR	0.871 (PVT1), 0.860 (GLIS2-AS1), for discriminating between PC patients and adenoma patients 0.950 (PVT1), 0.933 (GLIS2-AS1), for discriminating PCs with CDC73 mutation from wild-type carcinomas	[46]

↑, increase in lncRNA expression levels; ↓, reduction in lncRNA expression levels.

**Table 3:** Summary of differentially expressed circRNAs in parathyroid tumors.

Candidate circRNAs	Expression Levels	circRNAs Expression Profiling Platform	AUC Value	Study
cirRNA_051778, cirRNA_406174, cirRNA_0008267, circRNA_032603, circRNA_058097	↑ and ↓	Microarray, qPCR	/	[49]
circRNA_0017545, circRNA_0035563, circRNA_0075005, circRNA_0001687	↑	qPCR	0.770 (circRNA_0075005) for discriminating between PC patients and adenoma patients	[50]

↑, increase in circRNA expression levels; ↓, reduction in circRNA expression levels.

## Data Availability

Not applicable.
